# Low-SNR Infrared Point Target Detection and Tracking via Saliency-Guided Double-Stage Particle Filter

**DOI:** 10.3390/s22072791

**Published:** 2022-04-05

**Authors:** Liangjie Jia, Peng Rao, Yuke Zhang, Yueqi Su, Xin Chen

**Affiliations:** 1 Shanghai Institute of Technical Physics, Chinese Academy of Sciences, Shanghai 200083, China; jialiangjie@mail.sitp.ac.cn (L.J.); zhangyuke@mail.sitp.ac.cn (Y.Z.); suyueqi@mail.sitp.ac.cn (Y.S.); 2 Key Laboratory of Intelligent Infrared Perception, Chinese Academy of Sciences, Shanghai 200083, China; 3 University of Chinese Academy of Sciences, Beijing 100049, China

**Keywords:** infrared point target, target detection and tracking, particle filter, infrared remote sensing

## Abstract

Low signal-to-noise ratio (SNR) infrared point target detection and tracking is crucial to study regarding infrared remote sensing. In the low-SNR images, the intensive noise will submerge targets. In this letter, a saliency-guided double-stage particle filter (SGDS-PF) formed by the searching particle filter (PF) and tracking PF is proposed to detect and track targets. Before the searching PF, to suppress noise and enhance targets, the single-frame and multi-frame target accumulation methods are introduced. Besides, the likelihood estimation filter and image block segmentation are proposed to extract the likelihood saliency and obtain proper proposal density. Guided by this proposal density, the searching PF detects potential targets efficiently. Then, with the result of the searching PF, the tracking PF is adopted to track and confirm the potential targets. Finally, the path of the real targets will be output. Compared with the existing methods, the SGDS-PF optimizes the proposal density for low-SNR images. Using a few accurate particles, the searching PF detects potential targets quickly and accurately. In addition, initialized by the searching PF, the tracking PF can keep tracking targets using very few particles even under intensive noise. Furthermore, the parameters have been selected appropriately through experiments. Extensive experimental results show that the SGDS-PF has an outstanding performance in tracking precision, tracking reliability, and time consumption. The SGDS-PF outperforms the other advanced methods.

## 1. Introduction

Infrared point target (IRPT) detection and tracking is an important and challenging aspect of study in infrared remote sensing, which was widely used in both the civil and military fields [1,2,3,4,5]. For instance, space debris and failed satellites pose a serious threat to the security of spacecraft. In the shadowed regions, these targets have a very low quantity of radiant energy and are easily submerged in detector noise. Therefore, despite the very low background clutter of deep space, these targets were hardly detected due to the low signal-to-noise ratio (SNR) [6,7,8]. Furthermore, because of far-distance detecting and small target volume, these targets are less than one pixel on the focal plane and are known as the point target [9,10]. As a result, such targets are much harder to detect owing to the lack of texture and structural information [11,12,13]. In conclusion, it is worthwhile and challenging work to accurately detect and track the low-SNR IRPT in the deep space background.

The present infrared dim small target detection algorithms are emerging one after another. They can be divided into two major categories: detection before track (DBT) and track before detection (TBD). The DBT uses a single-frame image to detect the target. This kind of method depends greatly on the characters of the target and background, so strong noises and clutter have a negative impact on the detection ability. As for TBD, it uses multiple frames to track the target, which can suppress noise and clutter by using the temporal domain information. However, compared with DBT, TBD usually has poor real time. The following section will introduce the two methods in detail and our research motivation.

### 1.1. Detection before Track

Detection before track (DBT) methods extract the saliency of the target and the consistency of the background to detect the target by processing a single frame. According to the various technical approaches, DBT can be divided into three categories [14]: the background suppression-based methods, the human visual system-based methods, and the sparse and low-rank matrices recovery-based methods. Among them, the background suppression-based methods use various filters to predict and suppress the background. The Top-hat method [15], median filter [16], and facet model [17] are three of the most representative ones. As for the human visual system-based methods, these kinds of methods were inspired by the observation of the ways of humans, which uses the contrast between the target and background. The local contrast method [11] was proposed early. Lv et al. [18] proposed a neighborhood saliency characterization method, which can detect spatial weak point targets. An infrared small target detection method based on the multiscale local contrast measure using the local energy factor [19]was proposed to detect a small target with a complicated background. Among the sparse and low-rank matrices recovery-based methods, the image is viewed as a linear combination of the target, background, and noise. Based on the sparsity of the target and the low rank of the background, the targets are distinguished from the background through optimization. Non-convex optimization with the Lp-norm constraint (NOLC) method [14], low-rank representation (LRR) method [20], and non-convex rank approximation minimization joint l2,1 norm (NRAM) [21] represents good performance.

**Figure 1:**
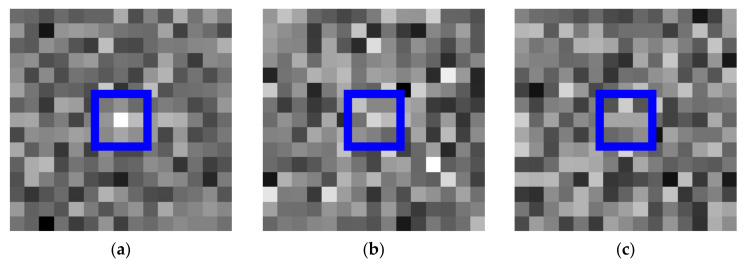
Infrared point target with different SNR, (**a**) SNR = 4; (**b**) SNR = 2; (**c**) SNR = 1.

Owing to the better performance in real time, DBT methods have received much attention and are widely used in practical applications. At present, DBT methods can detect the target in a complex moving background, such as intensive cloud edges, strong wind waves, and various ground objects. However, they may exhibit poor performance when the SNR is low. For a low-SNR image, the targets are submerged by sensor noise, which means the signal strength of the target is so weak that it cannot differentiate the noise in a single-frame image. As shown in Figure 1, we simulated three IRPTs with different SNRs, and the image size is 15 × 15 pixels. The IRPTs occupy 3 × 3 pixel units, which are pointed out by the blue box. In Figure 1a–c, the SNRs of the IRPTs are 4, 2, 1, respectively. The IRPT in Figure 1a is relatively obvious. However, the IRPT is very difficult to discover in Figure 1b,c. It is important to point out that, in this letter, the SNR is calculated by Equation (1):

(1) $$SNR=\frac{I-B}{\sigma_{n}},$$

where I is the signal strength of target center point, and B is the mean signal strength of background. The background means all the pixels of focal plane array. $\sigma_{n}$ represents the standard deviation of background.

### 1.2. Track before Detection

In view of the above-mentioned drawback of the DBT methods, the track before detection (TBD) methods were proposed, which use multiple frames to increase the energy of the target from the temporal domain. Presently, various TBD methods have been proposed, such as 3D matched filter [22,23,24], Hough transform [25,26], dynamic programming [27,28], particle filter (PF), and so on.

3D matched filter [22] is the earlier proposed TBD method, which accumulates the target signal in the space–time transformation domain. However, this method is not suitable for digital implementation because of much operation of the three-dimensional Fourier transform. Then, for addressing the problems above, the recursive moving-target-indication (RMTI) [23] was proposed. However, the application of these methods is limited because these methods depend on the velocity of the target to be known. Recently, Hou [24] et al. proposed a block-based improved RMTI algorithm to enhance the target energy in the velocity domain, which does not need the velocity of the target. The price is poor real time performance because this method matches velocity by traversing.

Hough transform [25] is proposed to extract the detection-tracks from the image. This method maps some potential targets to a specific Hough parameter space. In this Hough parameter space, the real targets can be accumulated, and the qualified tracks will be extracted easily. However, the original Hough transform must consume large amounts of storage resources to map all the potential targets, including the noise point. For addressing the problems above, the randomized Hough transform (RHT) [26] was proposed, which uses random sampling, converging mapping, and dynamic storage to avoid the drawback of the original Hough transform.

Dynamic programming [27] considers the accumulation energy of the target in a certain track as a decision function and considers the moving range of the target as a decision space. Then, the global optimized decision function can be obtained by recurrence, which is the target track. Then, for detecting a small dim target, a real-time visual enhancement method [28] is recommended to enhance the energy through dynamic programming. The visual quality of the target can be greatly improved.

Particle filter is a nonlinear and non-Gaussian estimation algorithm under the framework of Bayesian theory. TBD based on particle filter (PF-TBD) considers the problem of IRPT detection and tracking as a nonlinear and non-Gaussian problem. The state of the target can be estimated by particles with recursive filtering [29].

Compared with other TBD methods, the PF-TBD is simple to implement and has similar high accuracy with optimal estimation. Furthermore, the PF-TBD does not put constraints on the target motion and allows non-Gaussian dynamic noise and measurement noise. Nowadays, the PF-TBD is widely explored in low-SNR target detection and tracking.

Particle degradation has a negative impact on the detection accuracy of the PF-TBD. To address this problem, some studies have made great efforts regarding the resampling of particles. Long et al. [30]. proposed a TBD method based on multiple-model probability hypothesis density (MM-PHD), which has better performance in robustness and convergence speed. In the work of Zhang et al. [31], an intelligent PF method with a resampling of multi-population cooperation (RMPC-PF) divides particles into multiple populations to improve the particle diversity with a collaborative strategy. Moreover, Li et al. [32] established an adaptive strong tracking particle filter (AST-PF), which conducts the forgetting factor and the weakening factor to alleviate the degradation of PF.

The optimized distribution of the particles before resampling is also a great direction for enhancing the performance of the PF-TBD. Angel et al. [33] adopted a two-layer particle filter (TL-PF) to handle the track initiation and track maintenance, respectively. Chen et al. [34,35] introduced a bat algorithm and closed-loop control strategy into PF. The closed-loop control bat algorithm particle filter (CCBA-PF) performs well in low-SNR infrared target detection and tracking. In the work of Hu et al. [36], an improved PF based on an extended Kalman filter and genetic algorithm is proposed to solve the problem of particle degradation. Similarly, Havangi [37] proposed an improved unscented particle filter (IU-PF) to obtain optimization in proposal distribution and restrain the sample impoverishment. 

To accommodate the complex background, some studies improved the PF-TBD. Wang et al. [38] used a saliency appearance model and Eigen space model to suppress background clutter and enhance the accuracy of the target state estimation. These two complementary models were embedded in the particle filtering framework to track the maneuvering infrared target reliably. Kong et al. [39] used an 18-channel Gabor filter bank to extract the amplitude modulation (AM) features, which can distinguish the target from a complex background. Then, considering the observed kinematics of the target, the PF is adopted to suppress the false alarm. In the work of Zhang et al. [40], a target sparse representation model and constrained particle sampling model were introduced to enhance the tracking accuracy rate with a complex clutter background.

### 1.3. Motivation

As mentioned above, the PF-TBD has received much attention. However, the tracking accuracy and efficiency still have a much-enhanced space in low-SNR target tracking. Presently, the common proposal density is seriously affected by the intensive noise in the low-SNR image. As a result, a few particles are distributed in the target areas, which may lead to the false detection of the target even if many particles are used. Furthermore, intensive noise also has a bad influence on importance sampling. Owing to the target being buried by noise, targets may obtain very low weights in some frames. This will result in the loss of particles in the target area, which means that the PF-TBD will lose the target that has been tracked before.

Previous work has made a great deal of effort in proposal density optimization, overcoming sample impoverishment and avoiding particle degradation. The other recursive Bayesian filters is the most common improvement method for the importance sample of PF, for instance, extended Kalman filter [36] and unscented Kalman filter [37], etc. Furthermore, the random search methods, such as the bat algorithm [34,35] and genetic algorithm [36], are also used for optimizing the diversity of the particle states and overcoming the degradation defect of the particles. Although they improve the performance of PF estimation, these methods add serious algorithmic complexity. Besides, to address low-SCR target tracking, some methods strategically sample or resample particles to improve the particle diversity in a particular application. For instance, the Eigen space model [38] and saliency extraction [40] are used to limit the PF sampling process. Not only that: a circular collaborative structure [31] is proposed to optimize the resampling mechanism. As for robust tracking, two-layer PF [33] and backward recursion [30] are designed to improve the tracking performance. These methods both have an exclusive tracking mode and go into it when the newborn targets are detected.

Inspired by these improvement methods, we aim to design a saliency-guided double-stage particle filter (SGDS-PF) to address low-SNR target tracking. The SGDS-PF is divided into two modes: searching mode and tracking mode, which are composed of search PF and tracking PF, respectively. In searching mode, a multi-frame saliency extraction algorithm based on image patch is proposed to obtain high accuracy proposal density. Under the guidance of the optimized proposal density, a searching PF detects and outputs potential targets iteratively. Once a potential target has been detected, the particles of this potential target will go into tracking mode. In tracking mode, these potential targets will be continuously tracked by the tracking PF. Besides, a target confirmation algorithm is proposed to check potential targets. After multi-frame checking, the false targets will be eliminated. Conversely, the real targets will be locked, and their path will be outputted.

The main contributions of the letter are listed below.

1. Aiming at the poor particle sampling problem caused by low-SNR images, we proposed a multi-frame saliency extraction algorithm based on image patch. Unlike the traditional saliency extraction method using a single frame, a single-frame and multi-frame target accumulation method was designed to enhance the target and suppress noise first. On this basis, a likelihood estimation filter and image patch are used to extract target saliency and obtain a more accurate proposal density to guide the particles assigning.
2. A dual PF is given to handle the loss target problem caused by intensive noise in near real-time. The searching PF uses relatively few particles to detect targets roughly. Using very few particles, the tracking PF and the target confirmation algorithm further track and confirm targets. The fewer particles decide the low computational complexity and guarantee the near real-time. Furthermore, different from the traditional threshold segmentation, the guideline of the SGDS-PF is bold detection and cautious verification. Compared with the traditional method, the real targets masked by intensive noise will obtain more chances to be detected.
3. This letter provides the set value of key parameters by analyzing simulation experiments. Furthermore, a semi-physical simulating experiment using a real infrared camera was designed to verify the feasibility and robustness of this method.

The rest of this letter is organized as follows. In Section 2, the details of the SGDS-PF are covered. In Section 3, the experimental approaches, the set values of the main parameters, and the experimental results are shown. Then, Section 4 discusses the performances of the SGDS-PF and other PF methods. Finally, the conclusion of this letter is presented in Section 5.

## 2. Methodology

**Figure 2:**
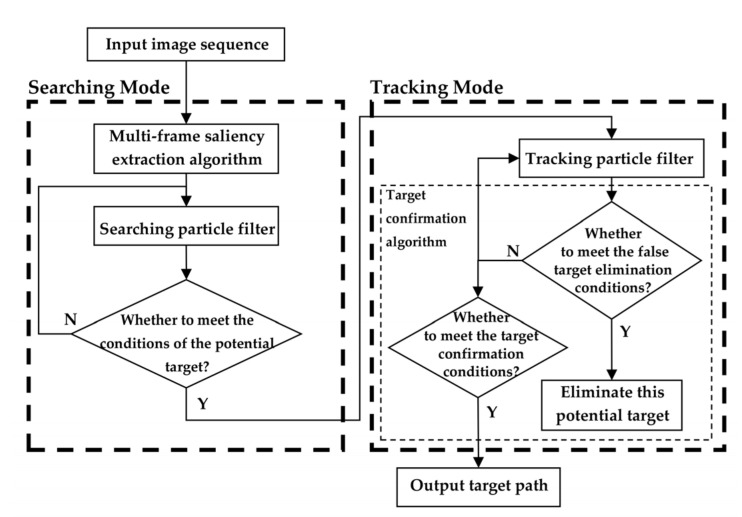
The block diagram of SGDS-PF.

Figure 2 is the block diagram of SGDS-PF, which is mainly divided into two modes, namely searching mode and tracking mode. In the searching mode, the multi-frame saliency extraction algorithm uses single-frame and multi-frame accumulation, likelihood estimation filter, and image patch to extract target saliency and obtain high quality proposal density. Guided by this proposal density, searching PF detects the potential targets and inputs them to tracking PF. In tracking mode, tracking PF combines with target confirmation algorithm to confirm whether the target is real or not. Then, the false target will be eliminated. In turn, tracking PF will keep tracking the others, and the real targets’ path will be output. In the rest of this section, we will introduce the modified PF of this letter, searching mode, and tracking mode in detail.

### 2.1. Modified Particle Filter

Consider a target with a certain intensity moving in the focal plane according to a nonlinear discrete system. First, the target dynamic model can be defined as:

(2) $$X_{f}=f\left(X_{f-1}\right)+V_{f},$$

(3) $$X_{f}=[x_{f}\;Vx_{f}\;y_{f}\;Vy_{f}\;I_{f}],$$

where *f* is the discrete-frame index and $X_{f}$ is the target state vector at frame *f*. In $X_{f}$, the $(x_{f}$,$y_{f})$, $(Vx_{f},Vy_{f}$), and $I_{f}$ represent the position, velocity, and intensity of target, respectively. $f\left(\cdot \right)$ is the target state transition function and $V_{f}$ is the process noise. Measured images are also recorded at discrete frame *f*. Measurement process is shown in (4)

(4) $$z_{f}=h\left(X_{f}\right)+W_{f},$$

where $z_{f}$ is the target measurement state vector at frame *f*, $h\left(\cdot \right)$ is the measurement function and $W_{f}$ is the measurement noise.

Then, the tracking problem can be formulated in the optimal estimation using recursive Bayesian theory. The formal recursive Bayesian solution can be presented as a two-step procedure, consisting of prediction and update. The prior probability density can be calculated by prediction procedure defined by:

(5) $$p(X_{f}|z_{1:f-1})=p(X_{f}|X_{f-1})p(X_{f-1}|z_{1:f-1}),$$

where $p(X_{f}|z_{1:f-1})$ is the prior probability density and $p(X_{f}|X_{f-1})$ is the transitional density that is defined by Equation (2). The update procedure uses the prior probability density and observation to derive posterior probability as follows:

(6) $$p(X_{f}|z_{1:f})=\frac{p(z_{f}|X_{f})p(X_{f}|z_{1:f-1})}{p(z_{f}|z_{1:f-1})},$$

where $p(z_{f}|X_{f})$ represents the similarity between the observed value and the transitional system state, which defined as likelihood, and $p(z_{f}|z_{1:f-1})$ is the normalization constant.

Theoretically, the posterior probability density can be calculated by Equations (5) and (6) now. However, the method cannot be applied to the type of moving target system directly due to the analytical solution of posterior distribution being hard to obtain. To address this problem, particle filtering is adopted. Particle filtering is the method that uses non-parametric Monte Carlo simulation methods to implement nonlinear and non-Gaussian recursive Bayesian filtering. Its main idea is using particles to sample and approximate posterior distribution.

In this letter, the basic concept follows the PF-TBD presented by Ristic and coworkers [41]. In this PF-TBD, target presence variable $E_{f}$ is modeled by a two-state Markov chain. $E_{f}$ can have 0 and 1. 0 represents a target is not present in this particle, and 1 represents the opposite. Based on the above statements, transitional probabilities of target “birth” ($P_{b}$) and “death” ($P_{d}$) can be defined as:

(7) $$P_{b}≜P\{E_{f}=1|E_{f-1}=0\},$$

(8) $$P_{d}≜P\{E_{f}=0|E_{f-1}=1\},$$

then, the probabilities of target stay alive and stay absent are defined as $1-P_{d}$ and $1-P_{b}$, respectively. On this basis, we introduce target presence count $Tp_{f}$ and particle population (PP) sequence $Seq_{k}$ into target state vector. Among them, target presence count $Tp_{f}$ denotes the number of times target existence, and PP sequence $Seq_{f}$ is the label of different PP. Now, the augmented state vector ($Y_{f}={\left[X_{f}{}^{T}\;E_{f}\;Tp_{f}\;Seq_{f}\right]}^{T}$) has eight components. The procedure of modified PF of this letter for TBD is presented as follows.

**Step 1**: Predict target existence variable $E_{f}^{l}$ of each particle (l=1, ⋯, L) using transitional probabilities of target $P_{b}$, $P_{d}$, $1-P_{b}$, and $1-P_{d}$. L is the number of particles.

**Step 2**: Predict target states of each particle that target present ($E_{f}=1)$. These particles can divide into two possible cases: newborn particles (from $E_{f-1}=0$ to $E_{f}=1$) and existing particles (from $E_{f-1}=1$ to $E_{f}=1$). For newborn particles, the target state is drawn as a sample from the proposal density. As for existing particles, the target state transforms by the target dynamic model that defined by Equation (2). In this letter, we adopt a nearly constant velocity model for target motion, which fits the application background. Hence, the Equation (2) can simplify as follows:

(9) $$Y_{f}=FY_{f-1}+V_{f},$$

(10) $$F=\left[\begin{array}{cccccccc} 1 & T & 0 & 0 & 0 & 0 & 0 & 0\\ 0 & 1 & 0 & 0 & 0 & 0 & 0 & 0\\ 0 & 0 & 1 & T & 0 & 0 & 0 & 0\\ 0 & 0 & 0 & 1 & 0 & 0 & 0 & 0\\ 0 & 0 & 0 & 0 & 1 & 0 & 0 & 0\\ 0 & 0 & 0 & 0 & 0 & 1 & 0 & 0\\ 0 & 0 & 0 & 0 & 0 & T & 1 & 0\\ 0 & 0 & 0 & 0 & 0 & 0 & 0 & 1 \end{array}\right],$$

where T denotes the frame period, normally T=1.

**Step 3:** Compute the importance weights of particle l by Equation (11)

(11) $${\overset{ˇ}{\omega }}_{f}^{l}=\left\{\begin{array}{cc} \prod_{i=1}^{In}\prod_{j=1}^{Im}\frac{p_{S+N}(z_{f}^{\left(i,j\right)}|X_{f})}{p_{N}\left(z_{f}^{\left(i,j\right)}\right)} & if\;E_{f}=1\\ 1 & if\;E_{f}=0 \end{array},\right.$$

where In and Im are the width and height of the image, respectively. Then, $p_{S+N}(z_{f}^{\left(i,j\right)}|Y_{f})$ is the probability density function (pdf) of target signal plus noise in pixel $\left(i,j\right)$, and $p_{N}\left(z_{f}^{\left(i,j\right)}\right)$ is the pdf of background noise in pixel $\left(i,j\right)$. They can be expressed as follows:

(12) $$p_{S+N}(z_{f}^{\left(i,j\right)}|Y_{f})=Ν\left(z_{f}^{\left(i,j\right)};h_{f}^{\left(i,j\right)},\sigma^{2}\right),$$

(13) $$p_{N}\left(z_{f}^{\left(i,j\right)}\right)=Ν\left(z_{f}^{\left(i,j\right)};0,\sigma^{2}\right),$$

here, $Ν\left(\cdot \right)$ is normal distribution function, and $h_{f}^{\left(i,j\right)}$ is the signal strength of the target at pixel $\left(i,j\right)$. In this letter, the point spread function is estimated by a two-dimensional Gaussian density with circular symmetry. Therefore, for a point target of intensity $I_{f}$ at position $\left(x_{f},y_{f}\right)$, the contribution to the pixel $\left(i,j\right)$ can be described by Equation (14).

(14) $$h_{f}^{\left(i,j\right)}\left(x_{f},y_{f}\right)\approx \frac{I_{f}}{2\pi \Sigma^{2}}exp\left[-\frac{{\left(i-x_{f}\right)}^{2}+{\left(j-y_{f}\right)}^{2}}{2\Sigma^{2}}\right],$$

where Σ is a parameter that represents the size of the dispersed spot. In application, this parameter is derived from the sensor and optical system. Furthermore, to reduce the computational load, the importance weight of a certain particle is only calculated in the 5 × 5 neighborhood of the particle, not the whole image as Equation (11). Therefore, according to Equations (11)–(14), the importance weights of particle l can be approximated as Equation (15).

(15) $${\overset{ˇ}{\omega }}_{f}^{l}=\prod_{i=i_{0}-2}^{i_{0}+2}\prod_{j=j_{0}-2}^{j_{0}+2}\exp\left[-\frac{h_{f}^{\left(i,j\right)}\left(h_{f}^{\left(i,j\right)}-2z_{f}^{\left(i,j\right)}\right)}{2\sigma^{2}}\right],$$

where $i_{0}$ and $j_{0}$ are the nearest integer value of particles’ x and y coordinates, respectively.

**Step 4**: Normalize the weight of particles by Equation (16)

(16) $$\omega_{f}^{l}=\frac{{\overset{ˇ}{\omega }}_{f}^{l}}{sum\left({\overset{ˇ}{\omega }}_{f}^{l}\right)},$$

**Step 5**: Resample the particles. The specific method is to stack the particle weights in order, as shown in Equation (17).

(17) $$\left\{\begin{array}{cc} S\omega_{f}^{1}=\omega_{f}^{1} & \\ S\omega_{f}^{l}=S\omega_{f}^{l-1}+\omega_{f}^{l} \end{array}\right.,$$

where $S\omega_{f}^{l}$ is the resampling interval value of the l-th particle. Then, as Equation (18) shows, generate L random numbers from 0 to 1 uniformly and randomly, and, if a random number falls into the resampling interval value of a certain particle, this particle will be copied except weight. Its weight will be assigned to 1/L.

(18) $$\left\{\begin{array}{c} Y_{f}^{\bar{l}}=Y_{f}^{l}\;if\;S\omega_{f}^{l-1}\leq U\left(0,1\right)<S\omega_{f}^{l}\\ \omega_{f}^{\bar{l}}=1/L \end{array}\right.,$$

where $U\left(\cdot ,\cdot \right)$ is the random uniform distribution function, and the two parameters are the upper and lower limits, respectively.

**Step 6**: Estimate target state by Equation (19)

(19) $${\hat{X}}_{f}=\frac{\sum_{l=1}^{L}X_{f}^{l}E_{f}^{l}}{\sum_{l=1}^{L}E_{f}^{l}},$$

The subsequent searching PF and tracking PF will be improved on the basis of the above procedure.

### 2.2. Searching Mode

**Figure 3:**
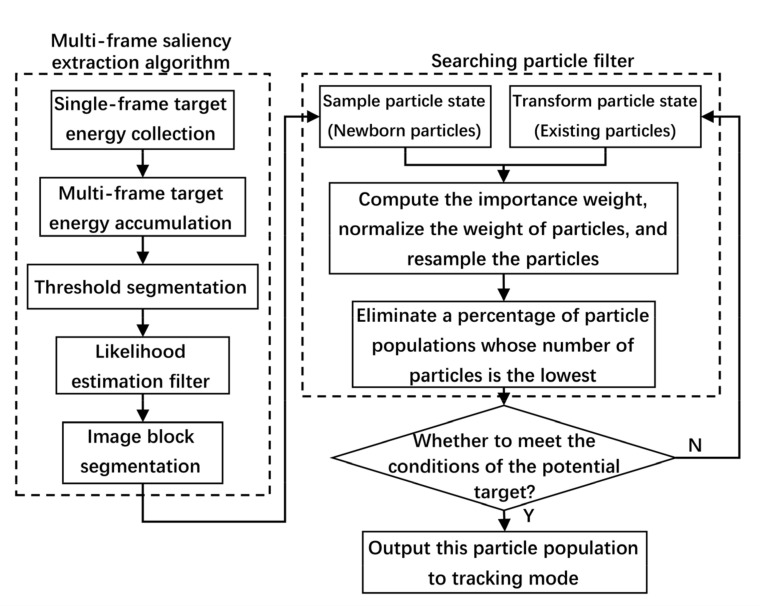
The block diagram of searching mode.

Searching mode is adopted to detect the potential targets roughly. In order to detect targets from intensive noise, the searching mode consists of the multi-frame saliency extraction algorithm and searching PF, as shown in Figure 3. Firstly, the multi-frame saliency extraction algorithm is used to enhance target and extract target saliency. Then, the potential targets are detected by searching PF. Finally, the potential targets and their particle states are output to tracking mode.

**1.**  **The multi-frame saliency extraction algorithm**

In order to overcome the intensive noise, target enhancement is an essential process. Therefore, before extracting saliency, we first enhance the target by single-frame target energy collection and multi-frame target accumulation. Single-frame target energy collection is to accumulate the energy in the 3 × 3 neighborhood, as shown in Equations (20) and (21). It is worth emphasizing that the size of neighborhood can modify according to the target size. In this letter, we mainly study point target, so we use 3 × 3 neighborhood here.

(20) $$I_{max}\left(i,j,f\right)=\max\left[I\left(i+m,j+n,f\right)\right]m,n=\left(-1,0,1\right),$$

(21) $$I_{se}\left(i,j,f\right)=\frac{I\left(i,j,f\right)\sum_{m=-1}^{1}\sum_{n=-1}^{1}I\left(i+m,j+n,f\right)}{I_{max}\left(i,j,f\right)},$$

where $I\left(i,j,f\right)$ is the signal strength of pixel $\left(i,j\right)$ at frame *f*. m and n are the offset of i and j, respectively, and their range of set values is [–1, 1]. Then, $I_{max}\left(i,j,f\right)$ denotes the max signal strength of neighborhood. Finally, $I_{se}\left(i,j,f\right)$ represents the result of single-frame enhancement, which collects neighborhood energy and balances it with the maximum value of the neighborhood.

**Figure 4:**
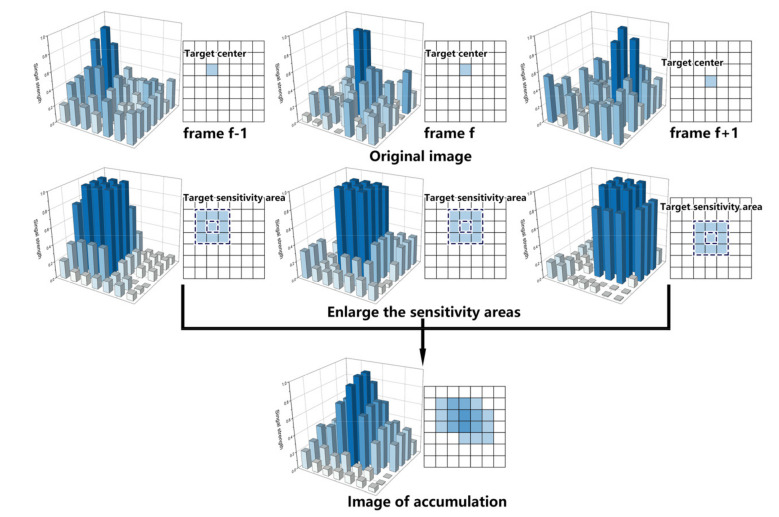
Schematic of single-layer multi-frame target accumulation.

**Figure 5:**
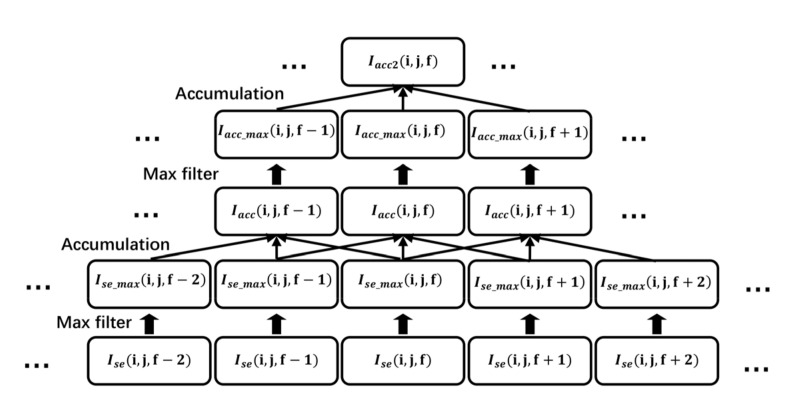
Schematic of two-layer multi-frame target accumulation.

After single-frame target enhancement, we proposed a two-layer multi-frame target accumulation to further enhance the target. In space target detection and tracking application, the distance between target and camera are over thousands of kilometers. Therefore, without loss of generality, we consider the velocities of most targets are usually no larger than a pixel per frame in the focal plane. Hence, we use the max filter defined by Equation (20) to enlarge the sensitivity areas of target and directly accumulate the adjacent frames to enhance the target energy. Figure 4 shows the single-layer multi-frame target accumulation. In Figure 4, the first-row images denote the input adjacent frame original images, and the second-row images are the images that have enlarged sensitivity area using the max filter. Obviously, the noise has been suppressed in the third-row enhanced image. In the first layer of max filter, the input is $I_{se}\left(i,j,f\right)$ and output is $I_{se\_max}\left(i,j,f\right)$. Then, directly accumulate the images of the three adjacent frames that have been filtered by the max filter as follows:

(22) $$I_{acc}\left(i,j,f\right)=\overset{k=1}{\underset{k=-1}{\sum }}\;I_{se\_max}\left(i,j,f+k\right),$$

where $I_{acc}\left(i,j,f\right)$ is the one-layer accumulation result and k is the frame offset. Then, using $I_{acc}\left(i,j,f\right)$ as the input, the above single-layer multi-frame target accumulation is repeated to obtain the second layer accumulation result as shown in Figure 5. Here, $I_{acc\_max}\left(i,j,f\right)$ represents the second layer max filter output, and $I_{acc2}\left(i,j,f\right)$ denotes the second layer accumulation result.

Significantly, in the low-SNR image, the max filter cannot promise to enlarge the sensitivity areas of the target at every frame because, in some frames, the target may be submerged by strong noise, which means that the signal strength of the target is not the largest in neighborhood. However, in these frames, most features of the target are also submerged. Namely, searching PF also hardly detects target using these frames. Furthermore, the target area output by multi-frame target accumulation is bigger than the real target. This is also a benefit to detect target by searching PF. The particles distribution is guaranteed to cover any direction of movement of the targets when the velocity of target is unknown.

After the target enhancement procedures above, a target segmentation using an adaptive threshold is adopted to roughly delimit the area of the target. The adaptive threshold is determined by Equation (23).

(23) $$T_{acc2}\left(f\right)=std2\left[I_{acc2}\left(i,j,f\right)]\left(2.2snr-2\right)+mean[I_{acc2}\left(i,j,f\right)\right],$$

where $std2\left(\cdot \right)$ and $mean\left(\cdot \right)$ are the standard deviation of image and average of image. snr is an input parameter, which is the lowest SNR of the target to be detected. This parameter can be estimated according to application. Then, the target segmentation is described by:

(24) $$I_{rt}\left(i,j,f\right)=\left[I_{acc2}\left(i,j,f\right)>T_{acc2}\left(f\right)\right]\&\&\left[I_{acc}\left(i,j,f\right)==I_{acc\_max}\left(i,j,f\right)\right],$$

where $I_{rt}\left(i,j,f\right)$ equal to 1 means the pixel $\left(i,j\right)$ at frame *f* may be a target. In turn, there is no target in this pixel at this frame.

After the steps above, the position of the particle distribution is confirmed. The likelihood estimation filter is proposed to calculate the eigenvalues of every pixel in the area of target. The eigenvalues can further guide the number of particles in each pixel in searching PF. Namely, the bigger the eigenvalues, the more particles. The essence of the likelihood estimation filter is calculating the importance weights of a fixed particle by estimating the signal strength. First, in likelihood estimation filter, the coordinates of particle are fixed in integer value. Therefore, for every particle’s 5 × 5 neighborhood, $h_{f}^{\left(i,j\right)}\left(x_{f},y_{f}\right)$ calculated by Equation (14) can be simplified as a five-by-five matrix as follows:

(25) $$h_{f}{}^{5\times 5}\approx I_{f}\frac{\exp\left\{-\left[\begin{array}{ccccc} 8 & 5 & 4 & 5 & 8\\ 5 & 2 & 1 & 2 & 5\\ 4 & 1 & 0 & 1 & 4\\ 5 & 2 & 1 & 2 & 5\\ 8 & 5 & 4 & 5 & 8 \end{array}\right]/2\Sigma^{2}\right\}}{2\pi \Sigma^{2}}=I_{f}D_{f}{}^{5\times 5},$$

where Σ is a fixed parameter as mentioned before. Therefore, this matrix only related to signal strength $I_{k}$, and we define the remainder as $D_{f}{}^{5\times 5}$. $I_{f}$ can be estimated by snr, which is the input parameter mentioned in Equation (23), as Equation (26) shows.

(26) $$I_{f}\in \left[\mathrm{mean}\left[I\left(f\right)\right]+\left(snr-2\right)\mathrm{std}2\left[I\left(f\right)],\mathrm{mean}[I\left(f\right)\right]+\left(snr+3\right)\mathrm{std}2\left[I\left(f\right)\right]\right],$$

Here, $I\left(f\right)$ denotes the origin image at frame *f*. Then, the importance weights calculated by Equation (15) can be derived as follows:

(27) $${\overset{ˇ}{\omega }}_{f}^{l}=exp\left[-\frac{sum\left[{\left(D_{f}{}^{5\times 5}\right)}^{2}\right]}{2\sigma^{2}}I_{f}{}^{2}+\frac{z_{f}^{\left(i,j\right)}*D_{f}{}^{5\times 5}}{\sigma^{2}}I_{f}\right],$$

where the part in $exp\left(\cdot \right)$ can be described by a quadratic equation with respect to variable $I_{f}$. Among them, the second-order coefficient is the same constant for every particle, and the one-order coefficient can be calculated by convolution, and the filter is $D_{f}{}^{5\times 5}$. Up to now, we could estimate the max importance weights for every pixel. For every pixel, there are three possible value points to obtain max importance weight, namely two boundary points of the range of signal strength defined in Equation (26) ($I_{f\_min}\left(i,j,f\right)$ and $I_{f\_max}\left(i,j,f\right)$) and the extreme value points ($I_{f\_ev}\left(i,j,f\right)$). Plug the three value points into Equation (27) and we can get three sets of corresponding importance weights: ${\overset{ˇ}{\omega }}_{f\_min}^{l}\left(i,j,f\right)$, ${\overset{ˇ}{\omega }}_{f\_max}^{l}\left(i,j,f\right)$, and ${\overset{ˇ}{\omega }}_{f\_ev}^{l}\left(i,j,f\right)$. Finally, the max importance weight of every pixel depends on its position of three value points, which is written as

(28) $${\overset{ˇ}{\omega }}_{f\_lef}^{l}\left(i,j,f\right)=\left\{\begin{array}{c} {\overset{ˇ}{\omega }}_{f\_min}^{l}\left(i,j,f\right)\;if\;I_{f\_min}\left(i,j,f\right)\geq I_{f\_ev}\left(i,j,f\right)\\ {\overset{ˇ}{\omega }}_{f\_max}^{l}\left(i,j,f\right)\;if\;I_{f\_max}\left(i,j,f\right)\leq I_{f\_ev}\left(i,j,f\right),\\ {\overset{ˇ}{\omega }}_{f\_ev}^{l}\left(i,j,f\right)\;\;\;\;\;\;\;\;\;\;others \end{array}\right.$$

where ${\overset{ˇ}{\omega }}_{f\_lef}^{l}$ denotes the max importance weight of every pixel estimated by likelihood estimation filter. Now, we define the whole saliency image as follows:

(29) $$Sal_{I}\left(i,j,f\right)={\overset{ˇ}{\omega }}_{f\_lef}^{l}\left(i,j,f\right)\cdot I_{rt}\left(i,j,f\right),$$

where $Sal_{I}\left(i,j,f\right)$ is the whole saliency image at frame *f*. The number of particles that can be allocated to pixel (i,j) is directly determined by $Sal_{I}\left(i,j,f\right)/sum\left[Sal_{I}\left(i,j,f\right)\right]$.

The last procedure of the multi-frame saliency extraction algorithm is image block segmentation. Theoretically, the number of false targets detected by target segmentation is inversely proportional to the image area. Therefore, there are so many false alarms in the very low-SNR and great-area image, which will divide the number of particles of the real targets. Hence, if the image area gets shrunk by double, the number of false alarms will compress by double too. Inspired by this, we introduce an image block segmentation here. First, the maximum image block side length $L_{ib}\left(snr\right)$ can be obtained by a lookup table. Here, this lookup table will be covered in Section 3.2, and the input parameter snr has been mentioned in Equation (23). Then, the number of segmentations of width and height are $In/L_{ib}\left(snr\right)$ and $Im/L_{ib}\left(snr\right),$ respectively. Here, · is the round-up function. In and Im are the width and height of the whole image, respectively. Finally, we eliminate the low-weight pixels of every segmented image block to optimize the particle diversity of searching PF. Assume that the b-th segmented saliency image block is denoted as $Sal_{I}{}^{b}\left(i,j,f\right)$. $N_{eff}^{b}$ is the number of effective particles in b-th segmented saliency image block [42], as Equation (30) shows.

(30) $$N_{eff}^{b}=\frac{1}{sum\left\{{\left[\frac{Sal_{I}{}^{b}\left(i,j,f\right)}{sum\left(Sal_{I}{}^{b}\left(i,j,f\right)\right)}\right]}^{2}\right\}},$$

Sort the $Sal_{I}{}^{b}\left(i,j,f\right)$ in descending order by every pixel’s value to get the weight set $W_{f}^{b}$. Therefore, the elimination of low-weight pixels is computed by Equations (31) and (32).

(31) $$T_{ep}^{b}=W_{f}^{b}N_{eff}^{b},$$

(32) $$OPsal_{I}{}^{b}\left(i,j,f\right)=\left\{\begin{array}{c} Sal_{I}{}^{b}\left(i,j,f\right)\;if\;Sal_{I}{}^{b}\left(i,j,f\right)\geq T_{ep}^{b}\;\\ 0\;\;\;\;\;\;if\;Sal_{I}{}^{b}\left(i,j,f\right)<T_{ep}^{b} \end{array}\right.,$$

where $T_{ep}^{b}$ is the threshold of eliminating low-weight pixels. $OPsal_{I}{}^{b}\left(i,j,f\right)$ is the optimized b-th segmented saliency image block, which has eliminated the low-weight pixels. Finally, $OPsal\_N_{I}{}^{b}\left(i,j,f\right)$ is the normalized $OPsal_{I}{}^{b}\left(i,j,f\right),$ as shown in Equation (33).

(33) $$OPsal\_N_{I}{}^{b}\left(i,j,f\right)=\frac{OPsal_{I}{}^{b}\left(i,j,f\right)}{sum\left(OPsal_{I}{}^{b}\left(i,j,f\right)\right)},$$

**Figure 6:**
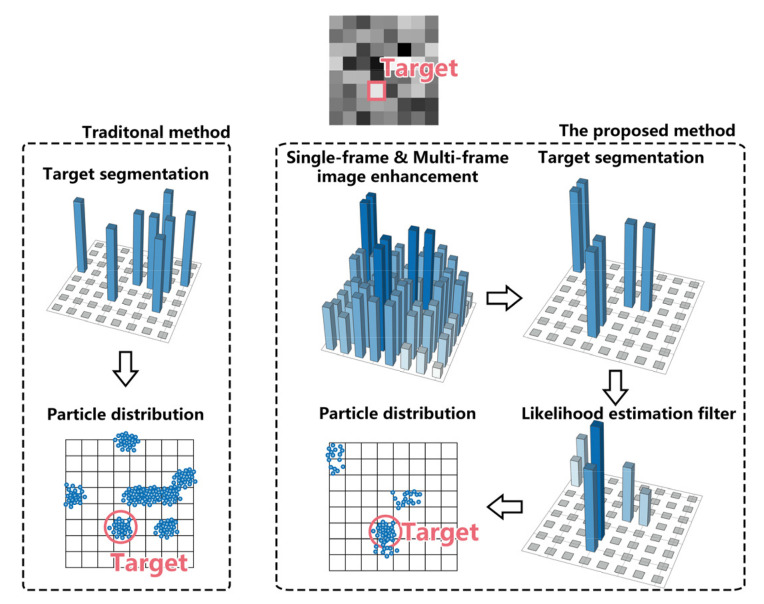
Structure flow chart of multi-frame saliency extraction algorithm.

The flow of the multi-frame saliency extraction algorithm is summarized in Figure 6. It can be seen that, by comparing the traditional method with the proposed method, the proposal density, namely particle distribution, is efficient and accurate.

**2.**  **Searching particle filter**

The segmented saliency image blocks have assigned a higher eigenvalue to the target and suppress noise in most frames. Searching PF is adopted to detect potential targets. The main process of searching PF is the same as Section 2.1 mentioned. Only improve at the beginning and the end; namely, the distribution of particles needs to be modified and a step to eliminate some particles after resampling particles needs to be added. It should be emphasized that each segmented saliency image block is searched by an independent searching PF. For instance, the b-th segmented saliency image block is searched by the b-th searching PF.

In the distribution of newborn particles, assume that the number of particles to be distributed in the b-th searching PF is $N_{dp}{}^{b}\left(f\right)$ at frame *f*. Therefore, the number of particles to be distributed in pixel $\left(i,j\right)$ is $OPsal\_N_{I}{}^{b}\left(i,j,f\right)\cdot N_{dp}{}^{b}\left(f\right)$. In the initial distribution of particles, $N_{dp}{}^{b}\left(f\right)$ is equal to the $N_{sump}^{b}$, which is the total number of particles in the b-th searching PF, and the value of $N_{sump}^{b}$ will be discussed in Section 3.2. Additionally, the state of particles is distributed as shown in Equation (34).

(34) $$\left\{\begin{array}{c} x_{f}\left(i,j,f\right)=i+U\left(-0.5,0.5\right)\\ Vx_{f}\left(i,j,f\right)=V\cdot \cos\left[U\left(0,2\pi \right)\right]\\ y_{f}\left(i,j,f\right)=j+U\left(-0.5,0.5\right)\\ Vy_{f}\left(i,j,f\right)=V\cdot \sin\left[U\left(0,2\pi \right)\right]\\ I_{f}\left(i,j,f\right)=U\left[I_{f}\left(f\right)\right]\\ E_{f}\left(i,j,f\right)=\left[U\left(0,1\right)<\mu \right]\\ Tp_{f}\left(i,j,f\right)=1\\ Seq_{f}\left(i,j,f\right)=CD_{8}\left[OPsal_{N}{{}_{I}}^{b}\left(i,j,f\right)>0\right] \end{array}\right.,\;V=U\left(V_{limit}\right)$$

where μ denotes the initial target existence probability, and $V_{limit}$ is the range of the target speed, which can be estimated according to the actual application. In addition, $CD_{8}\left(\cdot \right)$ is the eight-connected domain labeling function of binary graph. This function labels the serial numbers of the eight connected domains of the binary graph in order. In the subsequent distribution of particles, particles to be distributed are the newborn particles and the initial particles. Among them, the newborn particles are created in the prediction of target existence, and the initial particle is to supplement the particle eliminated in last iteration. The number of particles to be distributed is calculated by Equation (35).

(35) $$N_{dp}{}^{b}\left(f\right)=P_{e}\cdot N_{sump}^{b}+N_{nbp}{}^{b}\left(f\right),$$

where $P_{e}$ is the proportion of particles to be eliminated in each iteration. This parameter usually takes a value between 0.05 and 0.15 in this letter. $N_{nbp}{}^{b}\left(f\right)$ is the number of newborn particles. As for the state of particles, the distribution of variables except $E_{f}$ and $Seq_{f}$ is same as the first time. In the prediction of target existence variable, the newborn particles have been predicted such that their $E_{f}$ is equal to 1. Therefore, the $E_{f}$ of newborn particles is directly assigned to 1. On the other hand, the part of eliminated particles still adopts Equation (34) to update the $E_{f}$. As far as $Seq_{f}$ is concerned, this variable is used to label different PPs searching different target areas. Therefore, if there is already a PP searching in a certain target area, then the new particles to be distributed in the same area should be assigned the same PP sequence. Namely, add these new particles in this PP as shown in Equation (36).

(36) $$Seq_{f}\left(i,j,f\right)=\left\{\begin{array}{c} P_{pp}^{b}\left(i,j,f-1\right)\;\;if\;P_{pp}^{b}\left(i,j,f-1\right)\neq 0\\ N_{pp}^{b}+CD_{8}\left[OPsal_{N}{{}_{I}}^{b}\left(i,j,f\right)>0\right]\;others \end{array}\right.,$$

where $P_{pp}^{b}\left(\cdot ,\cdot ,\cdot \right)$ is the index image of PP sequence. $P_{pp}^{b}\left(i,j,f\right)$ denotes the PP sequence number of pixels $\left(i,j\right)$ at frame *f*. When $P_{pp}^{b}\left(i,j,f\right)$ is equal to 0, it means that pixel $\left(i,j\right)$ is no PP distribution at frame *f*. In addition, $N_{pp}^{b}$ represents the number of PPs in the b-th searching PF.

In traditional PF, the particles are only updated by the prediction of particle target states. This random iteration cannot eliminate some low weight PPs immediately, which may result in a larger PP size and has a negative influence on operation efficiency. Furthermore, more targeted elimination of particles can provide more particles for the next saliency image, which may include the target. Therefore, we introduce a step to eliminate particles after resampling particles. After resampling particles, the number of particles of PP represents the sum of importance weights of this population. Hence, first, sort the sum of particle number of PPs in ascending order to obtain $W_{pp}^{b}\left(p\right)$. Here, p is the index number of PP at block b. Then, stack $W_{pp}^{b}$ in order, as follows:

(37) $$\left\{\begin{array}{c} SW_{pp}^{b}\left(p\right)=W_{pp}^{b}\left(p\right)/N_{sump}^{b}\;\;\;\;\;if\;p=1\\ SW_{pp}^{b}\left(p\right)=SW_{pp}^{b}\left(p-1\right)+W_{pp}^{b}\left(p\right)/N_{sump}^{b}\;others \end{array}\right.,$$

**Figure 7:**
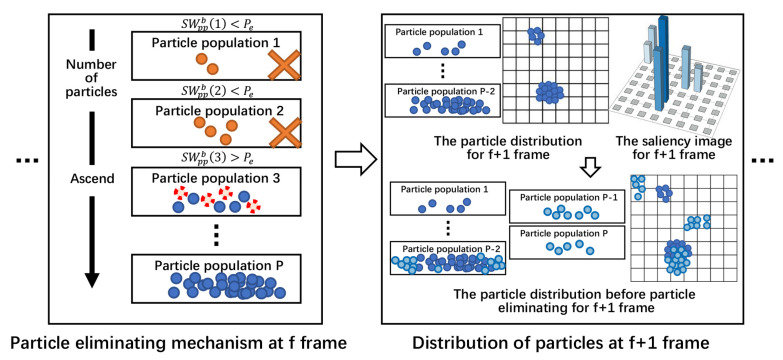
The distribution of particles and particle eliminating mechanism of searching PF.

Finally, eliminate the PPs and their particles in order until $SW_{pp}^{b}\left(p\right)$ is greater than or equal to $P_{e}$. Meanwhile, the p-th PP also should eliminate a part of particle randomly to maintain the particle number of each image block equal to $N_{sump}^{b}\left(1-P_{e}\right)$. Therefore, the number of particles to be eliminated is equal to $N_{sump}^{b}\cdot \left[SW_{pp}^{b}\left(p\right)-P_{e}\right]$. Figure 7 shows the distribution of particles and particle eliminating mechanism of searching PF.

As for now, the searching PF has completed one iteration. In order to prepare for the next iteration and the identification of potential targets, the state vector of each PP should be updated. The PP state vector was defined as $PY_{f}$ (${\left[PX_{f}{}^{T}\;PTp_{f}\;PSeq_{f}\;PE_{f}\;\right]}^{T}$), where $PX_{f}{}^{T}$ is the target state vector of PP, which is calculated by Equation (19), and $PTp_{f}$ is the average value of $Tp_{f}$ of particles, namely the average number of frames target existence in the PP. Then, $PE_{f}$ is the posterior probability of target existence of PP defined in Equation (38).

(38) $$PE_{f}=\frac{\sum_{pp}E_{f}}{N_{sump}^{b}},$$

where Npp is the number of particles in this PP, and $\sum_{pp}\cdot$ means the sum of a vector of the PP. Finally, $PSeq_{f}$ denotes the PP sequence number. This parameter is assigned in order from 1 to P for each PP. P is the total number of PPs. Meanwhile, the index image of PP sequence is updated here according to $PX_{f}{}^{T}$ and $PSeq_{f}$, which will be used by Equation (36) in next iteration.

In the identification of potential targets, the state vector of a PP should satisfy the two conditions defined below to declare that this PP has detected a potential target. These two conditions are listed below: (1) the posterior probability of target existence of PP needs to be greater than the threshold ($T_{PE}$) [41]; (2) the average number of frames target existence in the PP to be greater than the threshold ($T_{PTp}$), which can avoid the false alarm in the initial state. Finally, the PP detected as potential target and its particles will be eliminated from searching PF and input to the tracking PF. The pseudocode of a single cycle of the searching PF is presented in Algorithm 1.

**Algorithm 1**. Searching particle filter for a segmented image block.**Input:**$OPsal\_N_{I}{}^{b}\left(i,j,f\right)$ The normalize b-th segmented saliency image block**Output:**$PY_{f}$ and $Y_{f}$
 The particle population that detects the potential target and the particles of this particle population**1:** Predict target existence variable using transitional probabilities of target**2:** Calculate the number of particles to be distributed using Equation (35)**3:** Distribute newborn particles and initial particles using Equations (34) and (36)**4:for** n = 1: N **do****5:**  **if**
$E_{f-1}^{n}=1$ && $E_{f}^{n}=1$
**do****6:**    Transform the target state of particles using Equations (9) and (10)**7:**  **end if****8:**  Evaluate importance weight using Equations (11) and (15)
**9:**
**end for**
**10:** Normalize the weight of particles by Equation (16)**11:** Resample the particles using Equations (17) and (18)**12:** Sort the sum of particle number of particle populations in ascending order to get
$W_{pp}^{b}$**13:** Stack $W_{pp}^{b}$ to get $SW_{pp}^{b}$ using Equation (37)**14:for** p = 1: P **do****15:**  **if**
$SW_{pp}^{b}\left(p\right)<P_{e}$
**do****16:**    Eliminate this particle population and its particles**17:**    P = P − 1**18:**  **end if**
**19:**  **if**
$SW_{pp}^{b}\left(p\right)\geq P_{e}$
**do****20:**    Eliminate a part of particle of this particle population randomly
**21:**    break**22:**  **end if**
**23:**
**end for**
**24:**
**for** p = 1: P **do****25:**  Update the state vector $PY_{f}\left(p\right)$ using Equations (19) and (38).**26:**  **if**
$PTp_{f}\left(p\right)>T_{PTp}$ and $PE_{f}\left(p\right)>T_{PE}$**27:**    **output**
$PY_{f}\left(p\right)$ and $Y_{f}$**28:**    Eliminate this particle population and its particles**29:**  **end if**
**30:**
**end for**
**31:** Update the index image of particle population

### 2.3. Tracking Mode

Tracking mode consists of two main steps: tracking PF and target confirmation algorithm, as shown in Figure 2. Owing to the interference of intensive noise, the normal PF is easy to lose track of the target. Therefore, the tracking PF is proposed to keep tracking potential targets. Meanwhile, as mentioned above, the potential targets detected by the searching mode have false alarms. The target confirmation algorithm is adopted to eliminate false alarms and output the real target tracks.

**1.**  **Tracking PF**

Each potential target has its PP and particles, obtained from searching PF. It is stipulated that each potential target can have $N_{tpf}$ particles in the tracking PF. After random eliminating or random copying a certain number of particles, each potential target PP is independently iterated in different tracking PF. The main process of tracking PF is also the same as mentioned in Section 2.1. The difference is that the tracking PF does not have proposal density to guide the distribution of the newborn particles. The newborn particles are randomly copied from the other existing particles of its PP. The intent is to keep tracking potential targets by preventing newborn particles from scattering. After all PF steps, the target state of each PP calculated by Equation (19) will be saved as follows:

(39) $$Path^{p}\left(f\right)={\hat{X}}_{f},$$

where $Path^{p}\left(\cdot \right)$ records all the target states since the p-th PP is inputted in tracking PF, namely path information. Finally, PPs with their particles and path information would be input to the target confirmation algorithm to identify false or real targets.

**2.**  **Target confirmation algorithm**

As mentioned above, the tracking PF just tracks and updates the state of potential targets. A confidence evaluation mechanism based on the max importance weight and standard deviation of particles distribution is introduced to determine the real and false targets. The max importance weight is the character of intensity, and the deviation of particles distribution is the character of space. Ideally, particles that track real target will maintain high-level importance weight and focus tightly. On the contrary, if a certain PP is tracking a false target, its particles will be gradually dispersed because of resampling. Meanwhile, the importance weight of its particles will not be larger than the particles in search PF.

We use $C\left(p\right)$ to denote the confidence of the p-th PP. The value range is 0 to 1 and initial value is 1. In the character of intensity, assume that the max importance weight in searching PF at frame *f* is $MSW_{f}$, and $MTW_{f}\left(p\right)$ denotes the max importance weight of the p-th PP in tracking PF at frame *f*. By comparing the above two max importance weights, we can obtain the confidence evaluation factor (CEF) of intensity as follows:

(40) $$ICEF=\left\{\begin{array}{c} \frac{MTW_{f}}{MSW_{f}}\;if\;\frac{MTW_{f}}{MSW_{f}}\geq 1\\ 1\;if\;0.1\leq \frac{MTW_{f}}{MSW_{f}}<1\\ 0.9\;if\;\frac{MTW_{f}}{MSW_{f}}<0.1 \end{array}\right.,$$

In the character of space, the standard deviations of particle distribution in three directions, namely horizontal, vertical, and diagonal, are calculated by Equation (41).

(41) $$\left\{\begin{array}{c} dev_{x}^{p}=\sqrt{\frac{\sum_{n}^{N_{tpf}}{\left(x_{f}^{n}-\bar{x_{f}}\right)}^{2}}{N_{tpf}}}\\ dev_{y}^{p}=\sqrt{\frac{\sum_{n}^{N_{tpf}}{\left(y_{f}^{n}-\bar{y_{f}}\right)}^{2}}{N_{tpf}}}\\ dev_{xy}^{p}=\sqrt{\frac{\sum_{n}^{N_{tpf}}\left(x_{f}^{n}-\bar{x_{f}}\right)\left(y_{f}^{n}-\bar{y_{f}}\right)}{N_{tpf}}} \end{array}\right.,$$

where $\left(x_{f},y_{f}\right)$ is the position of particles of p-th PP. Then, we introduce the CEF of space to update the confidence in each iteration, as shown in Equation (42).

(42) $$SCEF\left(p\right)=\left\{\begin{array}{c} 1.1\;if\;dev_{x}^{p}\leq T_{devr}\;and\;dev_{y}^{p}\leq T_{devr}\;and\;dev_{xy}^{p}\leq T_{devr}\\ \min\left[\left(\frac{dev_{x}^{p}}{T_{devr}}\right),\left(\frac{dev_{y}^{p}}{T_{devr}}\right),\left(\frac{dev_{xy}^{p}}{T_{devr}}\right)\right]\;\;\;\;\;\;others \end{array}\right.,$$

where $T_{devr}$ is the threshold of standard deviation. The PP in a certain direction will be identified as diffuse if the standard deviation of this direction is larger than the threshold of standard deviation. $T_{devr}$ is determined by 3-sigma guidelines. We consider that, if 90% of the particles are within a certain pixel radius, the PP has not diffused. This pixel radius can be adjusted from 1.5 to 2.5 according to the actual application. For instance, if the pixel radius is equal to 2, $T_{devr}=2/1.3=1.5385$. As long as PP diffuses in any direction, it is considered that the PP diffuses, and the SCEF is set to the smallest ratio of the standard deviation in each direction to the standard deviation threshold. Conversely, the SCEF is equal to 1.1 to increase confidence. Finally, the confidence is updated by multiplying CEFs, as shown in Equation (43).

(43) $$C\left(p\right)=\left\{\begin{array}{c} 1\;if\;C\left(p\right)\cdot ICEF\left(p\right)\cdot SCEF\left(p\right)\geq 1\\ C\left(p\right)\cdot ICEF\left(p\right)\cdot SCEF\left(p\right)\;\;others \end{array}\right.,$$

Obviously, when a certain PP is tracking a false target, the confidence of this PP will inevitably continue to decline in the long run. Hence, we introduce a threshold to identify false target and denote it as $T_{ft}$. If $C\left(p\right)\;$ is smaller than $T_{ft}$, the p-th PP and its particles will be eliminated. As for tracking real targets, the confidence of PP will fluctuate between less than 1 and equal to 1. Therefore, we use a counter ($N_{rt}\left(p\right)$) to count the number of times confidence is equal to 1. Meanwhile, a threshold denoted as $T_{rt}$ is introduced to measure real target. If $N_{rt}\left(p\right)$ is larger than $T_{rt}$, the potential target of the p-th PP will be identified as the real target and its path will be output until it is lost. If $C\left(p\right)\;$ is not smaller than $T_{ft}$ and the p-th PP has been identified as real target, the path information of this PP will be outputted. The pseudocode of a single cycle of the tracking mode is presented in Algorithm 2.

**Algorithm 2**. Tracking particle filter for a potential target and target confirmation algorithm.**Input:**$PY_{f}$ and $Y_{f}$ The particle population that detects the potential target and the $N_{tpf}$ particles of this particle population**Output:**$Path^{p}$ The path information of real target**1:** Predict target existence variable using transitional probabilities of target**2:** Distribute newborn particles by randomly copying other particles of same PP**3:for** n = 1: $N_{tpf}$
**do****4:**  **if**
$E_{f-1}^{n}=1$ && $EE_{f}^{n}=1$
**do****5:**    Transform the target state of particles using Equations (9) and (10)**6:**  **end if****7:**  Evaluate importance weight using Equations (11) and (15)
**8:**
**end for**
**9:** Normalize the weight of particles by Equation (16)**10:** Resample the particles using Equations (17) and (18)**11:** Estimate target state of this particle population by Equation (19)**12:** Save target state in path information as Equation (39)**13:** Calculate the confidence evaluation factors ICEF and SCEF using Equations (40)–(42)**14:** Update the confidence of the p-th PP $C\left(p\right)$ using Equation (43)
**15:**
**if**

$C\left(p\right)<T_{ft}$

**do**
**16:**  Eliminate this particle population, its particles, and its path information**17:**  **return****18:else if** this particle population has been marked as real target**19:**  **Output** the path information of this particle population
**20:**
**end if**

**21:**
**if**

$C\left(p\right)=1$

**do**
**22:**  $N_{rt}\left(p\right)=N_{rt}\left(p\right)$ +1**23:**  **if**
$N_{rt}\left(p\right)>T_{r}$
**do****24:**    Mark this particle population as real target**25:**    **Output** the path information of this particle population**26:**  **end if**
**27:**
**end if**


## 3. Experimental Result and Discussion

### 3.1. Experimental Setting

In this letter, the simulation data were used to analyze key parameters and quantificationally verify the effectiveness of the SGDS-PF. Meanwhile, semi-physical simulation was adopted to verify the effectiveness and robustness of the SGDS-PF in the real shots.

In the simulation data, a nearly constant velocity model for target motion was used, and a stochastic model for target intensity [34,35,41] was introduced as follows:

(44) $$T_{f}=\mathrm{FT}+n_{f},$$

(45) $$F=\left[\begin{array}{ccccc} 1 & T & 0 & 0 & 0\\ 0 & 1 & 0 & 0 & 0\\ 0 & 0 & 1 & T & 0\\ 0 & 0 & 0 & 1 & 0\\ 0 & 0 & 0 & 0 & 1 \end{array}\right],$$

(46) $$Q=\left[\begin{array}{ccccc} \frac{q_{1}}{3}T^{3} & \frac{q_{1}}{2}T^{2} & 0 & 0 & 0\\ \frac{q_{1}}{2}T^{2} & q_{1}T & 0 & 0 & 0\\ 0 & 0 & \frac{q_{1}}{3}T^{3} & \frac{q_{1}}{2}T^{2} & 0\\ 0 & 0 & \frac{q_{1}}{2}T^{2} & q_{1}T & 0\\ 0 & 0 & 0 & 0 & q_{2}T \end{array}\right],$$

where $n_{f}$ is zero-mean white Gaussian noise with covariance Q. Besides, $q_{1}$ and $q_{2}$ denote the target state noise in target motion and intensity. As for sensor mode, point spread function is estimated by a two-dimensional Gaussian density with circular symmetry, as mentioned in Equation (14). After being modeled as above, some sequences can be generated with the following parameter: fuzzy parameters of the sensor Σ=0.7, background noise in each pixel with zero-mean white noise, whose variance σ=1, sampling period T=1, the level of target state noise $q_{1}=0.001$ and $q_{2}=0.01$. The initial intensity of the target is adjusted according to the required simulated SNR. The image size is 200 × 200 pixels and 50 × 50 pixels, which can verify the image segmentation block and consume as little computing time as possible for multiple experiments. The corresponding frame number is set to 70 frames and 150 frames to ensure that the target appears in most of the image frames.

**Figure 8:**
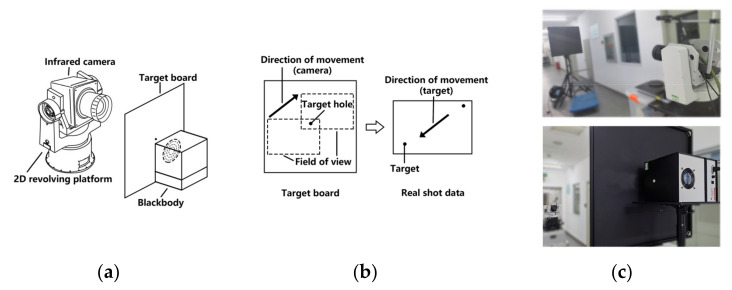
Semi-physical simulation method, (**a**) experimental equipment; (**b**) the target motion schematic; (**c**) experimental field.

Simulation data are an ideal experimental data, which do not consider the influence of nonuniformity, blind pixels, and pixel format. However, the real infrared images of space point targets are difficult to obtain. Especially, the real paths of the space targets are needed to verify the accuracy of the methods. Therefore, to verify the robustness of the SGDS-PF, a semi-physical simulation method was designed, as shown in, to obtain the real shot data. Figure 8a shows the experimental equipment. Target board and blackbody were used to semi-physically simulate IRPT, and 2D revolving platform controlled the movement of the infrared camera. Through moving the infrared camera, the static IRPT can move in field of view, as shown in Figure 8b. Figure 8c shows the actual experimental field. The parameters of the infrared camera used in the semi-physical simulation experiment are listed in Table 1.

Finally, the SGDS-PF compares the original PF [41], the closed-loop control bat algorithm particle filter (CCBA-PF) [35], and the intelligent particle filter with resampling of multi-population cooperation (RMPC-PF) [31]. Methods are implemented under MATLAB R2018a with an Intel Core 2.80 GHz processor and 8 GB of physical memory. 

### 3.2. Parameters Analysis

In the SGDS-PF, several key parameters are vital to detecting and tracking performance. These key parameters are the maximum image block side length ($L_{ib}$), the number of particles in each searching PF ($N_{sump}^{b}$), the thresholds of identifying the potential target ($T_{PTp}$ and $T_{PE}$), the number of particles in each tracking PF ($N_{tpf}$), and the thresholds of identifying real targets and false targets ($T_{rt}$ and $T_{ft}$).

**Figure 9:**
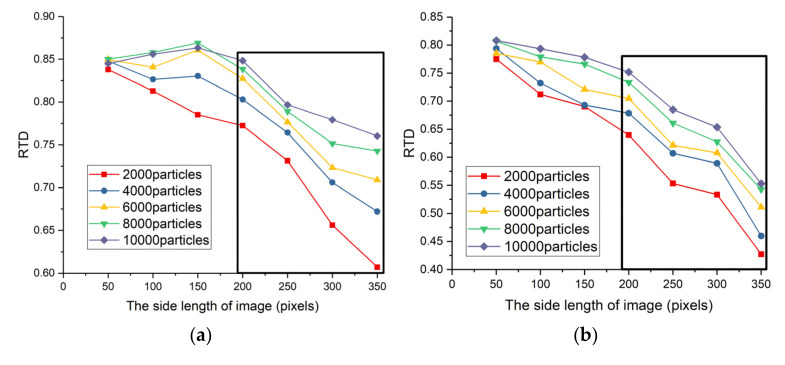

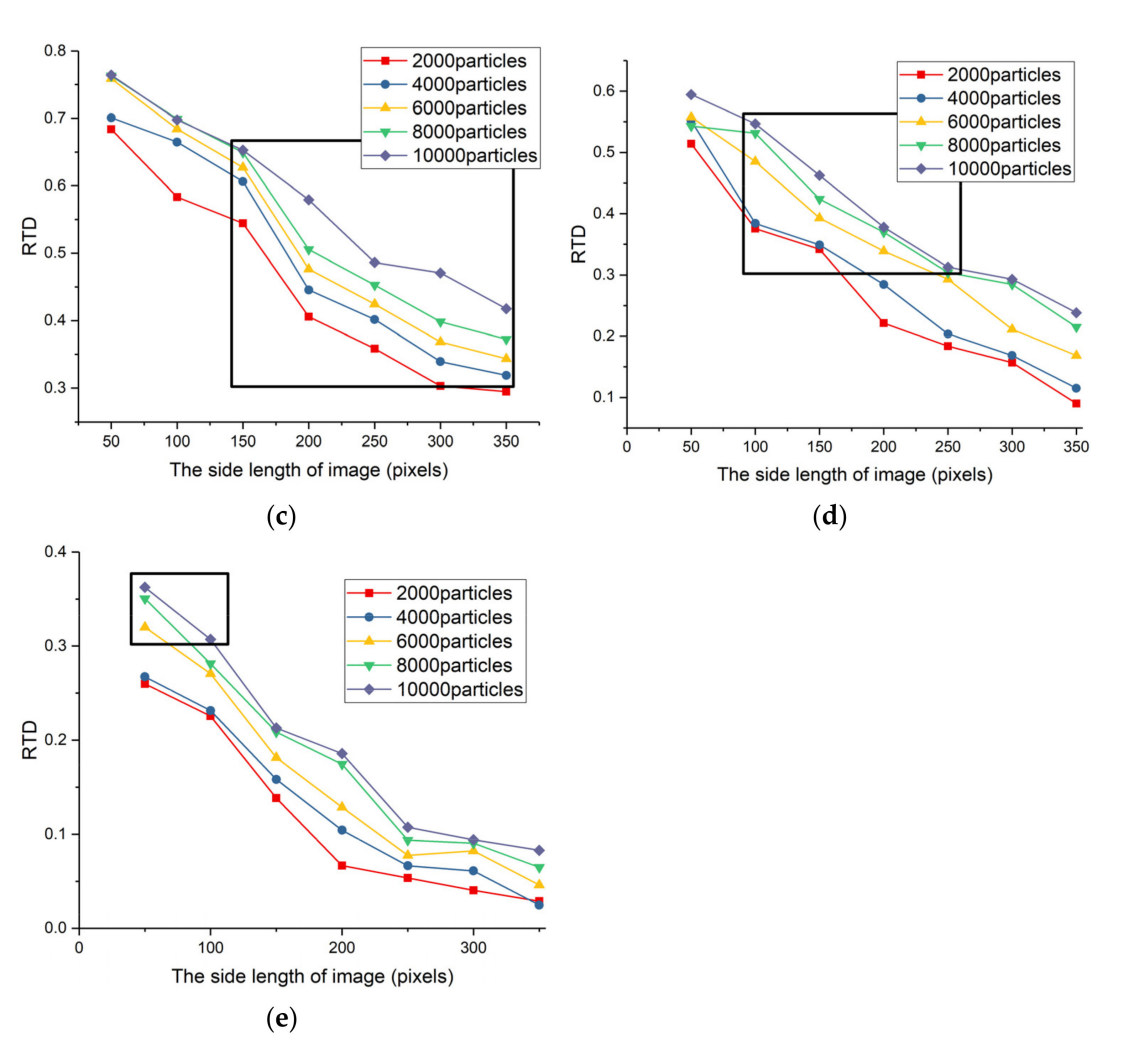
The searching ability of searching PF for different image size, SNR, and number of particles, (**a**) SNR = 2; (**b**) SNR = 1.8; (**c**) SNR = 1.6; (**d**) SNR = 1.4; (**e**) SNR = 1.2.

The process parameters ($L_{ib}$ and $N_{sump}^{b}$) of searching PF directly influence its searching ability. We introduce the rate of target detected (RTD) to evaluate the searching ability of searching PF. The RTD indicates the ratio of target detection frames to target existence frames. Figure 9 shows the searching ability of searching PF. The horizontal axis of Figure 9 is the side length of the square simulation image, indicating the image size, and the vertical axis of Figure 9 is the RTD. The larger the RTD, the stronger the searching ability.

Obviously, smaller image blocks and more particles can obtain better performance in SGDS-PF. However, it also means larger computing resources consumption and poor real-time. Furthermore, more particles receive limited improvement performance in small image blocks. For instance, the RTD gap is not very large when $L_{ib}$ is smaller than 200, as shown in Figure 9a,b. However, in Figure 9d,e the RTD is in a fearful recession as the SNR declines and image area increases. Therefore, it is necessary to assign proper particles in proper image block to keep the searching ability. Considering the real-time and detection performance, the value range of $L_{ib}$ and $N_{sump}^{b}$ in different SNRs is marked by the black box in Figure 9.

**Figure 10:**
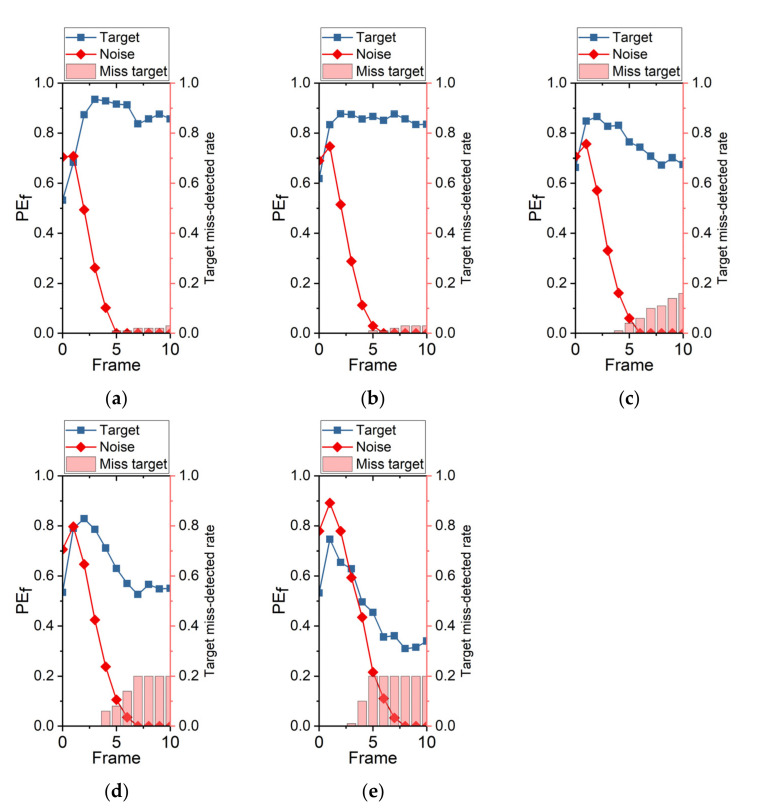
$PE_{f}$ comparison with target and noise, (**a**) SNR = 2; (**b**) SNR = 1.8; (**c**) SNR = 1.6; (**d**) SNR = 1.4; (**e**) SNR = 1.2.

In order to obtain accurate detection results of searching mode, we compared the posterior probability of target existence of PP ($PE_{f}$) and target miss-detected rate, as shown in Figure 10, to select proper thresholds ($T_{PE}$ and $T_{PTp}$). The searching mode of SGDS-PF was used to detect the targets in simulation images with different SNRs. Then, the $PE_{f}$ of targets and noise were recorded at each frame. The average $PE_{f}$ of the target and the largest first-percentile $PE_{f}$ of noise are plotted in Figure 10. In other words, the $PE_{f}$ of 99 percent of noise stays below the red line of Figure 10. Obviously, the $T_{PE}$ should be larger than the $PE_{f}$ of noise and less than the $PE_{f}$ of target at each frame. Meanwhile, the target miss-detected rate was introduced to consider the lost targets with the enhancement of frames. As shown in Figure 10e, if $T_{PTp}$ is larger than 5, the 20 percent targets will be excluded in this stage. However, the $PE_{f}$ of target is smaller than the $PE_{f}$ of noise when $T_{PTp}$ is smaller than 5. Hence, the performance envelope of our searching mode is SNR larger than 1.2. The larger the distance between the $PE_{f}$ of target and the $PE_{f}$ of noise, the easier it is to identify the target from noise. It can be seen from Figure 10a–d that $T_{PTp}$ can be set to 4 to 5 considering the lower target miss-detected rate and larger value space of $T_{PE}$. Owing to the suppression of false alarms in tracking mode, the $T_{PE}$ only needs to be slightly larger than the $PE_{f}$ of noise to further ensure the target detection rate. Consequently, the $T_{PE}$ is set to 0.1 to 0.2.

**Figure 11:**
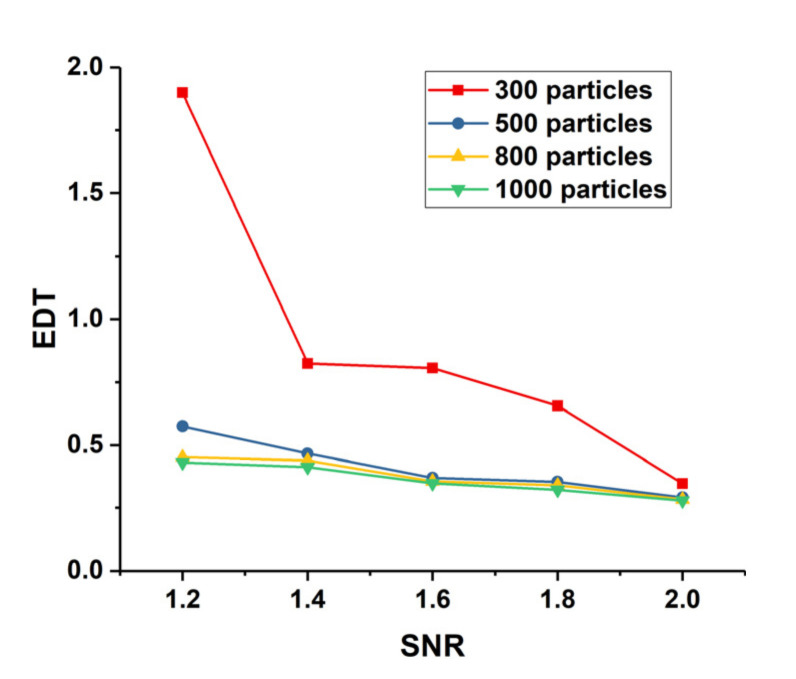
The tracking ability of tracking PF with different number of particles.

The number of particles in tracking PF determine the performance of locking target. Owing to initial of searching mode, the difference of tracking targets is only influenced by the SNR of image. Therefore, we use the Euclidean distance between the estimated and actual situation of target to indicate the tracking performance as follows:

(47) $$EDT=\sqrt{{\left({\hat{x}}_{p}-x_{rt}\right)}^{2}+{\left({\hat{y}}_{p}-y_{rt}\right)}^{2}},$$

where $\left({\hat{x}}_{p},{\hat{y}}_{p}\right)$ is the estimated position of the targets by tracking PF, and $\left(x_{rt},y_{rt}\right)$ is the actual position of the targets. Figure 11 shows the tracking effect of the searching PF with different number of particles. Clearly, 300 particles cannot track target well when SNR is smaller than 2 compared with more particles. To save the computing resources, $N_{tpf}$ is set to 500; namely, 500 particles are distributed to each tracking PF.

**Figure 12:**
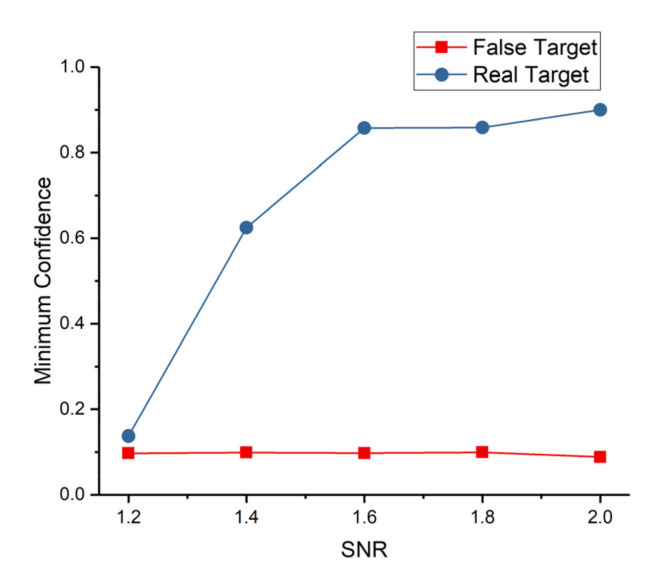
The minimum confidence of the real and false targets with different SNR.

**Figure 13:**
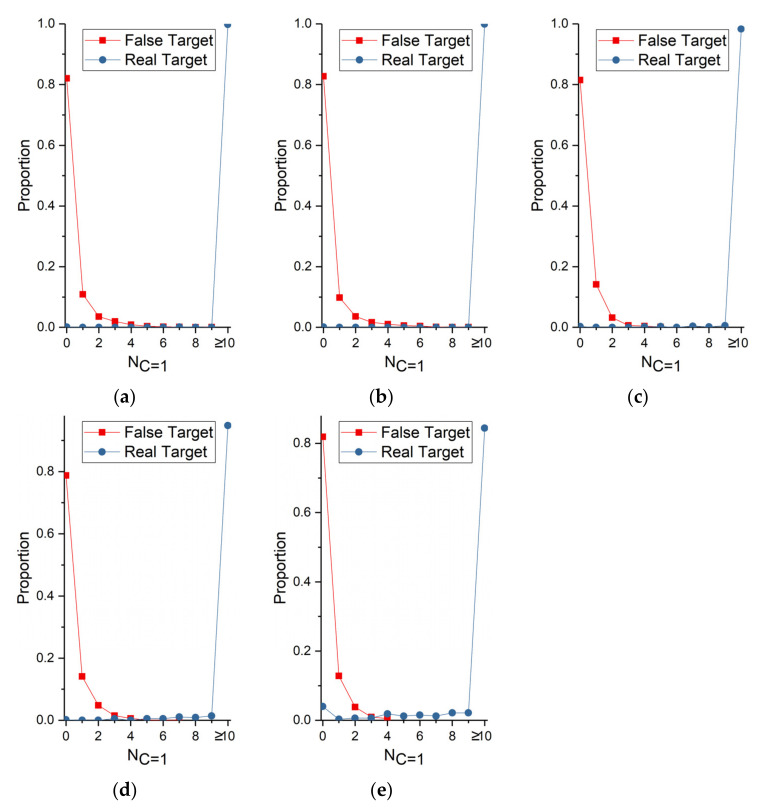
The proportion of $N_{C=1}$ of the real and false targets with different SNR, (**a**) SNR = 2; (**b**) SNR = 1.8; (**c**) SNR = 1.6; (**d**) SNR = 1.4; (**e**) SNR = 1.2.

Finally, the thresholds ($T_{ft}$ and $T_{rt}$) of identifying real targets and false targets have direct bearing on false alarm rate and detection rate. The minimum confidence (MC) of each real target and false target was recorded. The largest MC of false target and the smallest 10th-percentile MC of real target were plotted in Figure 12. The MC of 90 percent of real target is larger than the value of blue line and red line (the largest MC of false target) in Figure 12. Clearly, the confidence can easily distinguish the real target from false target when SNR is larger than 1.2. The $T_{ft}$ is set to 0.1 to 0.4. Meanwhile, the same conclusion with Figure 10 that the performance envelope of our searching mode is SNR larger than 1.2 is obtained. Furthermore, the number of times the real and false target confidence is equal to 1 ($N_{C=1}$) was recorded, and their proportion was plotted in Figure 13. This feature can effectively detect real targets at all tested SNRs. Over 80 percent of false target confidence never equals 1. Conversely, more than 85 percent of real target confidence is equal to 1 no less than 10 times. Consequently, $T_{rt}$ is set to 4 to 10.

The key parameters and thresholds of the SGDS-PF are obtained based on experiments and analysis. The suggested selection of these parameters and thresholds under different SNRs are listed in Table 2. It is important to note that these parameters and thresholds are upwardly compatible with a high SNR. In real application, the SNR of the target detected may be difficult to estimate. Then, the parameters and thresholds can be selected according to the standard of the minimum SNR (SNR = 1.4). However, the cost of this approach is to consume more computing resources.

### 3.3. Experimental Result

**Figure 14:**
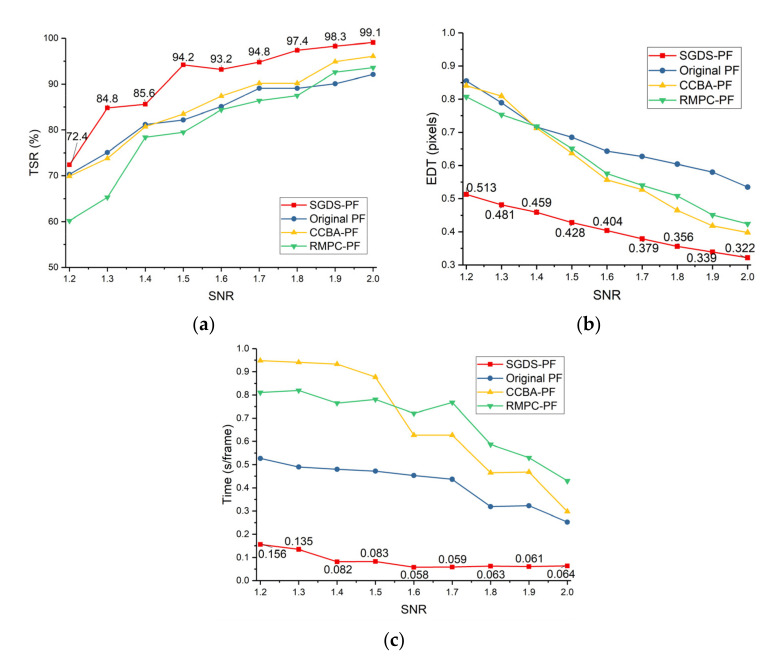
Performance comparison of methods in 50 × 50 pixels simulation image, (**a**) TSR; (**b**) EDT; (**c**) time consumption.

**Figure 15:**
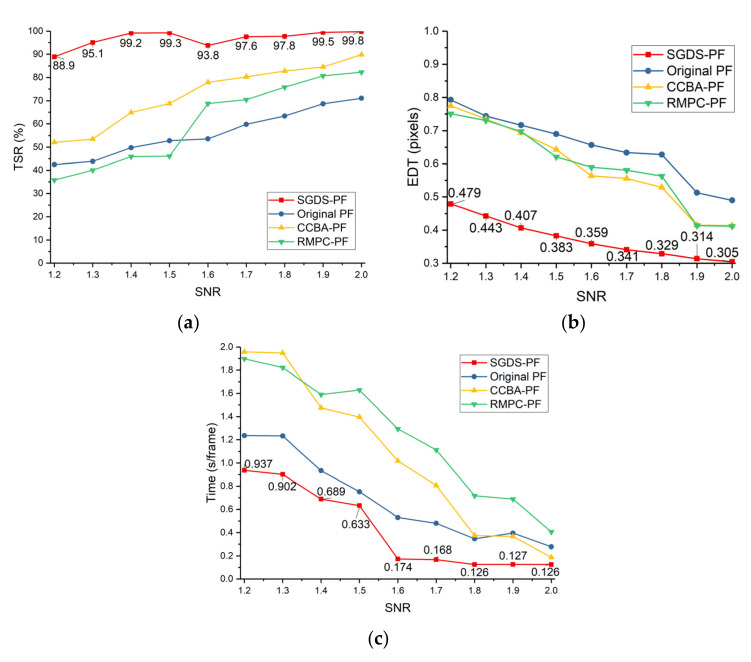
Performance comparison of methods in 200 × 200 pixels simulation image, (**a**) TSR; (**b**) EDT; (**c**) time consumption.

The simulation data (50 × 50 pixels, 70 frames and 200 × 200 pixels, 150 frames) with different SNRs (1.2~2.0) were used to test the different methods (SGDS-PF, original PF, CCBA-PF, and RMPC-PF). To measure the accuracy of methods, the EDT mentioned in Equation (47) was used again as a quantitative indicator. The smaller the EDT is, the more accurate the tracking results are. Furthermore, to give a tracking reliability evaluation of the methods, the detection success ratio (DSR) [40] and the tracking success ratio (TSR) were adopted as in Equations (49) and (49)

(48) $$DSR=\frac{N_{EDT<T_{0}}}{N_{tf}},$$

(49) $$TSR=\frac{\sum_{n}^{Ntest}(DSR_{n}>T_{1})}{Ntest}\times 100\%,$$

where $N_{EDT<T_{0}}$ means the number of acceptable tracking results. If the EDT is smaller than $T_{0}$ at a certain frame, the method can declare that it detected the target successfully at this frame. Then, $N_{tf}$ is the number of frames that exist for the target. Similarly, Ntest denotes the number of tests, and, if the rate of successful detection is not less than a certain percentage ($T_{1})$ in a certain test, the method can declare that the tracking was successful in this test. In this letter, $T_{0}$ is 2 pixels, and $T_{1}$ is 0.2. Thus, the EDTs, TSRs, and elapsed time per frame of methods using the simulation data with different SNRs are plotted in Figure 14 and Figure 15. Meanwhile, Table 3 shows the particle numbers and other parameters of the compared methods. The parameters of SGDS-PF have been listed in Table 2.

**Figure 16:**
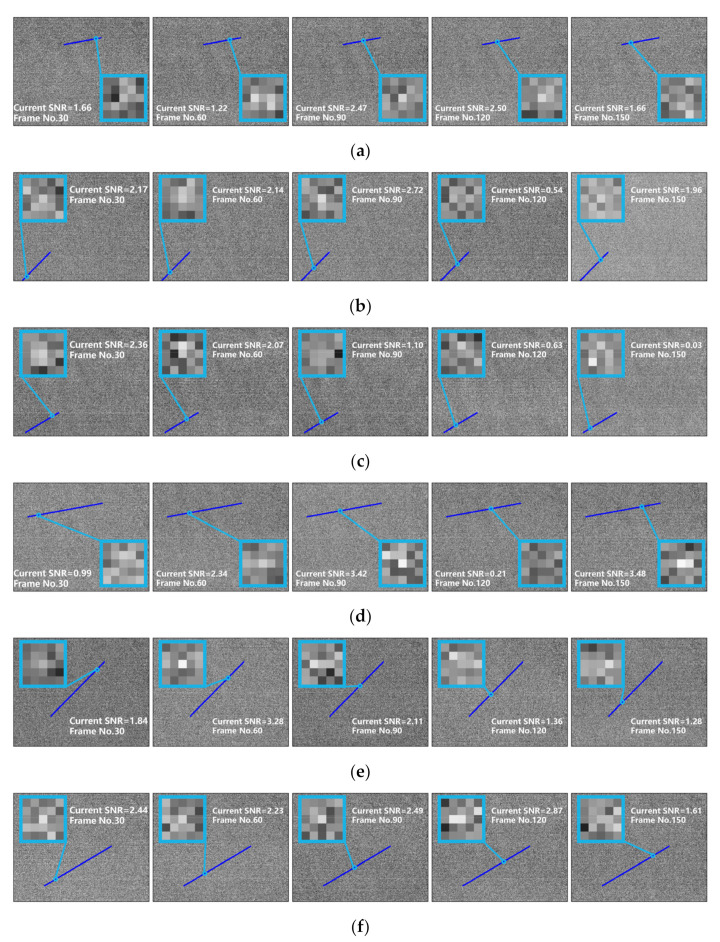
The semi-physical simulation data set (SNR = 2), (**a**) Seq.1 V = 0.5; (**b**) Seq.2 V = 0.5; (**c**) Seq.3 V = 0.5; (**d**) Seq.4 V = 1; (**e**) Seq.5 V = 1; (**f**) Seq.6 V = 1.

The semi-physical simulation dataset is divided into three different SNRs (1.2, 1.6, and 2.0). Each SNR is further divided into two categories according to the velocity of the target (0.5 pixels per frame and 1 pixel per frame). Besides, each sequence includes 200 frames. Figure 16 shows the semi-physical simulation dataset for SNR = 2. The dark blue lines are the target path, and the light blue box is the target at current frame. Without loss of generality, the semi-physical simulation datasets for SNR = 1.6 and SNR = 1.2 were obtained by adding Gaussian noise in the data for SNR = 2.

Significantly, the target paths in Figure 16 were extracted in the same motion data with high SNR. Therefore, these target paths do not reflect the precise location of the target in each frame. Hence, the EDT cannot be calculated. Furthermore, owing to the limited number of tests, the TSR has no reference. Finally, we choose the DSR and elapsed time to evaluate algorithm effectiveness, as listed in Table 4. Meanwhile, Table 5 shows the parameters of the compared methods. The parameters of the SGDS-PF were still set as in Table 2.

## 4. Discussion

The particle filter has been widely studied by researchers, and many improved methods based on PF were also proposed to detect and track the dim point target. At present, researchers pay more attention to the diversity optimization of particle states or the proposal density optimization regarding low SCR images. However, the low-SNR targets detection and tracking was ignored. Compared with original and recently enhanced PF, SGDS-PF has the superiority in tracking accuracy and time consumption.

The SGDS-PF uses the double-stage PF to extract the characters of targets and identifies potential targets and real targets, respectively. The key idea is bold detection and cautious verification, which reduces the missed detection and ensures accuracy. As shown in Figure 14a and Figure 15a, the SGDS-PF has the best TSR. Furthermore, in the bigger image size and longer frame length, the image block segmentation provides more opportunity to detect targets. For instance, compared with 50 × 50 simulation data, the TSR of the SGDS-PF increases even more in 200 × 200 simulation data when SNR equals 1.2 and 1.4. In addition, the tracking PF keeps the particles within a small potential target neighborhood. However, fewer particles compared to other methods are used in total. Increased particles per unit target area ensures the accuracy of the target tracking. Therefore, the SGDS-PF detects targets more accurately, as shown in Figure 14b and Figure 15b. Meanwhile, the searching PF uses a multi-frame accumulation and likelihood estimation filter to obtain more accurate proposal density in low-SNR images. Owing to the accurate proposal density, fewer particles are used in the SGDS-PF, which consumes fewer computational resources. Hence Figure 14c and Figure 15c show that the SGDS-PF obtains good real-time performance.

From the perspective of the diversity optimization of particle states, the RMPC-PF adopts multi-population cooperation to avoid decreasing particle diversity in the resampling stage. The results illustrate that the RMPC-PF obtains a more accurate estimated result than the original PF. However, the convergence speed of this method is slower than other methods because a part of the particles is assigned in other relatively high weight areas without a target. Especially in low-SNR images, the importance weights of the target’s area do not have distinct advantages. Therefore, the RMPC-PF is more likely to miss the target and has no advantage in computing resources. These defects are more notable in low-SNR images (SNR smaller than 1.6), as shown in Figure 14a and Figure 15a. As for the CCBA-PF, this method optimizes the particle distribution through the bat algorithm. Essentially, this method iteratively optimizes the state of each particle within a single frame. The tracking accuracy is better than the other methods except the SGDS-PF. However, this method of trading time for accuracy sacrifices real-time performance. Furthermore, owing to intensive noise, the CCBA-PF is easy to fall into a local optimum, which has a negative influence on the TSR, as shown in Figure 14a and Figure 15a. In the original PF, if there are enough particles, the original PF can achieve a good result. However, the computational complexity of the original PF is determined by the number of particles. Therefore, considering the real-time performance, the original PF can only use a limited number of particles and receive a similar result.

In semi-physical simulation experiments, the SGDS-PF still retains an obvious advantage over the other methods, as shown in Table 4. The SGDS-PF has good robustness for real shoot data when the SNR equals 1.6 and 2.0. However, the SGDS-PF fails to detect the target four times when the SNR is equal to 1.2. On the one hand, as mentioned above, the upper npimd of the SMR when using the SGDS-PF is 1.2. On the other hand, the SGDS-PF assigns particles according to the saliency of the targets. However, owing to the similar feature with the targets, the blind pixels might be assigned many particles in the low-SNR images. Therefore, real targets lost many particles, which had a negative influence on the target detection of the SGDS-PF. Furthermore, in the SGDS-PF, the images were segmented into six blocks when the SNR was equal to 1.2. Each image block was assigned to 6000 particles. The consumption of massive resources reduces the processing frequency of the SGDS-PF to 1 Hz. In conclusion, for the actual application of the SGDS-PF, the effective blind pixel suppression preprocess and parallel processing should be introduced to obtain better performance in low-SNR images.

## 5. Conclusions

In this letter, a saliency-guided double-stage particle filter was proposed for infrared point target detection and tracking. In the searching mode, a multi-frame saliency extraction algorithm based on an image patch was adopted for high accurate proposal density. Then, the searching PF detects potential targets using a few particles. In the tracking mode, the tracking PF uses even fewer particles to track and confirm the potential targets, respectively. Finally, the parameters and thresholds have been selected appropriately through experiments. In addition, the simulation data and semi-physical simulation real shoot data were obtained to verify the performance of the SGDS-PF. The extensive experimental results show that the SGDS-PF has an obvious advantage in tracking precision, tracking reliability, and time consumption. Moreover, for future actual applications, the SGDS-PF may obtain a better performance under effective blind pixels suppression and parallel processing.

## Figures and Tables

**Table 1:** Specifications of the infrared camera used to obtain experimental data.

Format	320 × 256
Pixel pitch	30 μm
Spectral Range	8–12 μm
F-number	2.0
Noise Equivalent Temperature Difference (NETD)	30 mk
Framerate	30 Hz
Bits per pixel	14 bits

**Table 2:** Typical selection of parameters and thresholds.

SNR	$\left[2\right.,\left.\infty \right)$	$\left[1.8\right.,\left.2\right)$	$\left[1.6\right.,\left.1.8\right)$	$\left[1.4\right.,\left.1.6\right)$	$\left(0\right.,\left.1.4\right)$
$L_{ib}$	200~350	200~350	150~350	100~250	50~100
$N_{sump}^{b}$	2000~6000	2000~6000	2000~6000	6000~10,000	6000~10,000
$T_{PE}$	0.1	0.1	0.1	0.2	0.2
$T_{PTp}$	5	5	5	4	4
$N_{tpf}$	500				
$T_{ft}$	0.6~0.8	0.4~0.6	0.4~0.6	0.2~0.4	0.1~0.12
$T_{rt}$	10~9	10~9	9~7	8~6	6~4

**Table 3:** The particle numbers and other parameters of the compared methods.

Compared Methods	Image Size	SNR				
		[2,∞)	[1.8,2)	[1.6,1.8)	[1.4,1.6)	(0,1.4)
Original PF	50 × 50	PN:10,000	PN:14,000	PN:18,000	PN:24,000	PN:30,000
	200 × 200	PN:14,000	PN:20,000	PN:25,000	PN:30,000	PN:60,000
CCBA-PF	50 × 50	PN:500 UIN:40	PN:800 UIN:40	PN:1100 UIN:40	PN:1500 UIN:40	PN:2000 UIN:40
	200 × 200	PN:500 UIN:80	PN:1000 UIN:80	PN:1800 UIN:80	PN:2700 UIN:80	PN:4000 UIN:80
RMPC-PF	50 × 50	PN:6000 PPN:3	PN:10,000 PPN:3	PN:14,000 PPN:3	PN:16,000 PPN:3	PN:18,000 PPN:3
	200 × 200	PN:6000 PPN:5	PN:10,000 PPN:5	PN:14,000 PPN:5	PN:18,000 PPN:5	PN:24,000 PPN:5

NOTES: PN denotes the particle number. PPN means the number of particle populations. UIN represents the upper limit of the iteration number.

**Table 4:** The DSR and elapsed time of methods tested in semi-physical simulation data set.

DSR (%)	Time (s/f)	SGDS-PF		Original PF		CCBA-PF		RMPC-PF	
SNR = 2	Seq.1	63.0	0.165	0	1.200	26.0	1.687	3.0	1.698
	Seq.2	87.4	0.156	78.9	1.159	83.7	1.712	66.8	1.723
	Seq.3	91.5	0.149	0	1.211	5.5	1.684	6.0	1.743
	Seq.4	74.0	0.144	0	1.109	3.5	1.657	0.5	1.687
	Seq.5	89.5	0.146	86	1.257	73.0	1.738	84.5	1.750
	Seq.6	81.5	0.150	76.5	1.186	10.5	1.708	39.5	1.645
SNR = 1.6	Seq.1	65	0.446	0	1.884	1.0	3.318	0.5	3.121
	Seq.2	83.7	0.454	0	1.823	7.4	3.360	0.5	3.014
	Seq.3	82.5	0.436	1.5	1.908	0	3.298	0	3.200
	Seq.4	40.0	0.427	0	1.837	0	3.259	0	2.989
	Seq.5	77.5	0.437	77.0	1.917	73.0	3.246	60.5	3.059
	Seq.6	75.5	0.435	0	1.857	0.5	3.354	0.5	3.078
SNR = 1.2	Seq.1	0	1.059	0	2.169	1.0	5.168	0.5	6.678
	Seq.2	0	1.104	0	2.213	0	5.218	1.6	6.753
	Seq.3	91.5	1.062	0.5	2.202	0	5.239	0	6.879
	Seq.4	0	1.070	0	2.187	0.5	5.173	0	6.701
	Seq.5	0	1.078	0	2.158	0.5	5.210	0	6.698
	Seq.6	76.5	1.017	64.5	2.189	66.5	5.187	68.0	6.715

**Table 5:** The particle numbers and other parameters of the compared methods.

Compared Methods	SNR					
	2		1.6		1.2	
Original PF	PN:40,000		PN:82,000		PN:123,000	
CCBA-PF	PN:4100	UIN:80	PN:8200	UIN:100	PN:12,000	UIN:120
RMPC-PF	PN:12,000	PPN:6	PN:41,000	PPN:6	PN:60,000	PPN:6

NOTES: PN denotes the particle number. PPN means the number of particle populations. UIN represents the upper limit of the iteration number.

## Data Availability

Not applicable.
